# Statin use in patients with hormone receptor‐positive metastatic breast cancer treated with everolimus and exemestane

**DOI:** 10.1002/cam4.5369

**Published:** 2022-10-19

**Authors:** Kyoungmin Lee, Eunjin Noh, Seok Joo Moon, Yoonjung Yoonie Joo, Eun Joo Kang, Jae Hong Seo, In Hae Park

**Affiliations:** ^1^ Division of Hemato‐Oncology, Department of Internal Medicine Korea University Guro Hospital, Korea University College of Medicine Seoul Republic of Korea; ^2^ Smart Healthcare Center Korea University Guro Hospital, Korea University College of Medicine Seoul Republic of Korea; ^3^ Institute of Data Science, Korea University Seoul Republic of Korea

**Keywords:** breast neoplasm, estrogen receptor, everolimus, exemestane, hydroxymethylglutaryl‐CoA reductase inhibitors

## Abstract

**Background:**

We analyzed the effect of statins in patients with hormone receptor‐positive (HR+) metastatic breast cancer treated with everolimus + exemestane (EverX).

**Materials and Methods:**

We conducted a nationwide retrospective cohort study using the National Health Insurance database with patients who received EverX for metastatic breast cancer between 2011 and 2019.

**Results:**

Of 224,948 patients diagnosed with breast cancer, 1749 patients who received EverX for at least 30 days were included. Among them, 500 (28.6%) patients were found to take statins with EverX treatment (statin group), and the median duration of this combination was 5.36 months. The median time to treatment duration (TTD) for EverX and the overall survival (OS) were significantly higher in the statin group than in the no‐statin group [7.69 vs. 5.06 months, *p* < 0.001; 45.7 vs. 26.0 months, *p* < 0.001, respectively]. Multivariable Cox analysis revealed that the use of statins was associated with prolonged TTD [HR = 0.67 (95% CI, 0.59–0.77)] and OS [HR = 0.57 (95% CI, 0.46–0.70)] for EverX even after adjustment for other covariates.

**Conclusion:**

Statins may have synergistic effects with endocrine therapy with the mTOR inhibitor everolimus, and improve survival in patients with HR+ metastatic breast cancer.

## Graphical abstract

Statins, commonly used to lower systemic cholesterol levels, lead to favorable survival outcomes in the treatment of patients with HR+ ABC. Statins may have synergistic effects with endocrine therapy with the mTOR inhibitor everolimus and may improve the treatment outcomes of EverX.

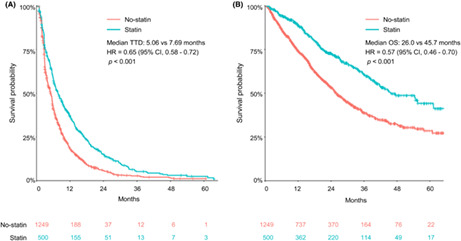

## 1 INTRODUCTION

Hormone‐receptor positive (HR+) breast cancer represents 60–65% of all breast malignancies, and the treatment for HR+ breast cancer has been focused on targeting the estrogen receptor (ER) signaling pathway.^1^ Cholesterol forms a biochemical scaffold for all steroid hormones, including estradiol, and it has therefore been thought to play a crucial role in HR+ breast cancer.^2^ Statins are cholesterol‐lowering HMG CoA reductase inhibitors that block the rate‐limiting step in cholesterol biosynthesis.^3^ In addition to cholesterol metabolism, statins have been suggested to reduce intracellular hormone production in breast cancer cells^4^ and the levels of circulating cholesterol and its metabolites, in particular 27‐hydroxycholesterol (27HC), which acts as a ligand of the ER.^5^, ^6^ In addition, many studies have confirmed that statins have pleiotropic effects beyond their lipid‐lowering properties, with a wide range of anti‐inflammatory, immunomodulatory, anti‐thrombotic, and anti‐tumor effects.^7^ One of the mechanisms of the anti‐tumor effects of statins was explained by the statin‐mediated inhibition of the mammalian target of rapamycin (mTOR) signaling.^7^, ^8^, ^9^, ^10^

mTOR is a serine/threonine protein kinase located downstream of the phosphatidylinositol 3‐kinase (PI3K)/AKT pathway.^11^ This is a key intracellular signaling system that drives cellular growth and survival, and emerging evidence indicates that hyperactivation of the PI3K/AKT/mTOR pathway is a key mechanism of endocrine resistance in HR+ breast cancer.^12^ Therefore, the mTOR inhibitor everolimus in combination with endocrine therapy has become a therapeutic option for HR+ advanced breast cancer (ABC) upon disease progression after first‐line anti‐hormonal therapies with or without CDK4/6 inhibitors.

In the Republic of Korea, combined treatment with everolimus and exemestane (EverX) is reimbursed by the National Health Insurance as a second‐line or higher‐order therapy in HR+ ABC. In this study, using the Korean National Health Insurance (NHI) and the Health Insurance Review and Assessment (HIRA) databases, we explored the synergistic effect of statins in patients with HR+ ABC treated with palliative endocrine therapy, especially EverX.

## 2 MATERIALS AND METHODS

### 2.1 Korean National Health Insurance big data

The NHI program of Korea is a universal health coverage system that covers almost the entire Korean population.^13^ All data on medical expenses collected during the reimbursement process of healthcare providers are stored and managed by the HIRA.^14^ The HIRA contains medical billing data including the number of visits, number of days spent in outpatient visits, number of prescriptions, details of medical treatment items, and details of prescriptions. The HIRA also contains information regarding demographic characteristics, medical history (treatments, procedures, and examinations), principal diagnosis, and comorbidities, which are coded using the International Classification of Diseases, 10th revision (ICD‐10).^14^, ^15^ Datasets stored in the data warehouse of the HIRA are sources for generating statistics on healthcare services, developing indices of quality for each type of care, and health research.^16^

Access to HIRA data is regulated by the Rules for Data Exploration and Utilization of the HIRA, and we used data with the approval of the HIRA data access committee. This study was approved by the Institutional Review Board of the Korea University Guro Hospital (IRB number 2020GR0282), and written informed consent was waived due to the nature of the study.

### 2.2 Study population

To identify patients diagnosed with breast cancer between January 2011 and October 2019, we extracted data of patients with ICD‐10 codes for breast cancer (C50) from January 2010 to October 2019 and excluded those who already had a breast cancer code during 2010. We then retrieved patients who were prescribed antineoplastic doses of everolimus (ingredient codes 485605ATB, 485606ATB, and 485607ATB) during the study period. Basic data on each patient's clinical information, including demographics, diagnosis of other comorbidities, prescribed drugs, the duration of each drug's prescription, and survival, were curated from the Healthcare Bigdata Hub under the HIRA. The ICD‐10 codes used to identify comorbidities such as hypertension, diabetes mellitus (DM), and hyperlipidemia were I10–I15, E10–E14, and E78, respectively. The ingredient codes used in this study for other drugs, including various types of statins, metformin, and chemotherapeutic agents, are described in Table S1. Statins and metformin were incorporated into our analyses regardless of whether they were the sole ingredient or one of several ingredients of the relevant drugs.

### 2.3 Statistical analysis

Qualitative or categorical variables were presented as frequencies and proportions and compared using the chi‐square test. Continuous variables were presented as medians with interquartile ranges (IQRs) and compared using the Student's *t*‐test. For evaluating the survival outcomes of EverX treatment, time to treatment duration (TTD) for EverX was estimated from the date of the first prescription of everolimus to the date of the last prescription, and overall survival (OS) was estimated from the date of the first prescription of everolimus to the date of death from any cause or the last date of the use of medical service. Data were censored if the patients were still on EverX treatment or were alive at the time of data analysis (October 31, 2019). Survival curves were estimated using the Kaplan–Meier method and compared using the log‐rank test. Univariable and multivariable Cox proportional hazard analyses were conducted to evaluate the impact of concomitant statins on treatment outcomes of EverX while adjusting for covariates, including age at which EverX treatment was started (< 60 or ≥ 60 years), duration of prior aromatase inhibitor treatment (≤6 months, 6 months–1 year, or >1 year), prior history of cytotoxic chemotherapy, comorbidities, and use of metformin.

All statistical analyses were performed using SAS version 9.4 (SAS Institute, Cary, NC, USA) and R studio version 3.5.1 (R Foundation for Statistical Computing, Vienna, Austria). Statistical significance was set at *p* < 0.05.

## 3 RESULTS

### 3.1 Patient characteristics

**FIGURE 1:**
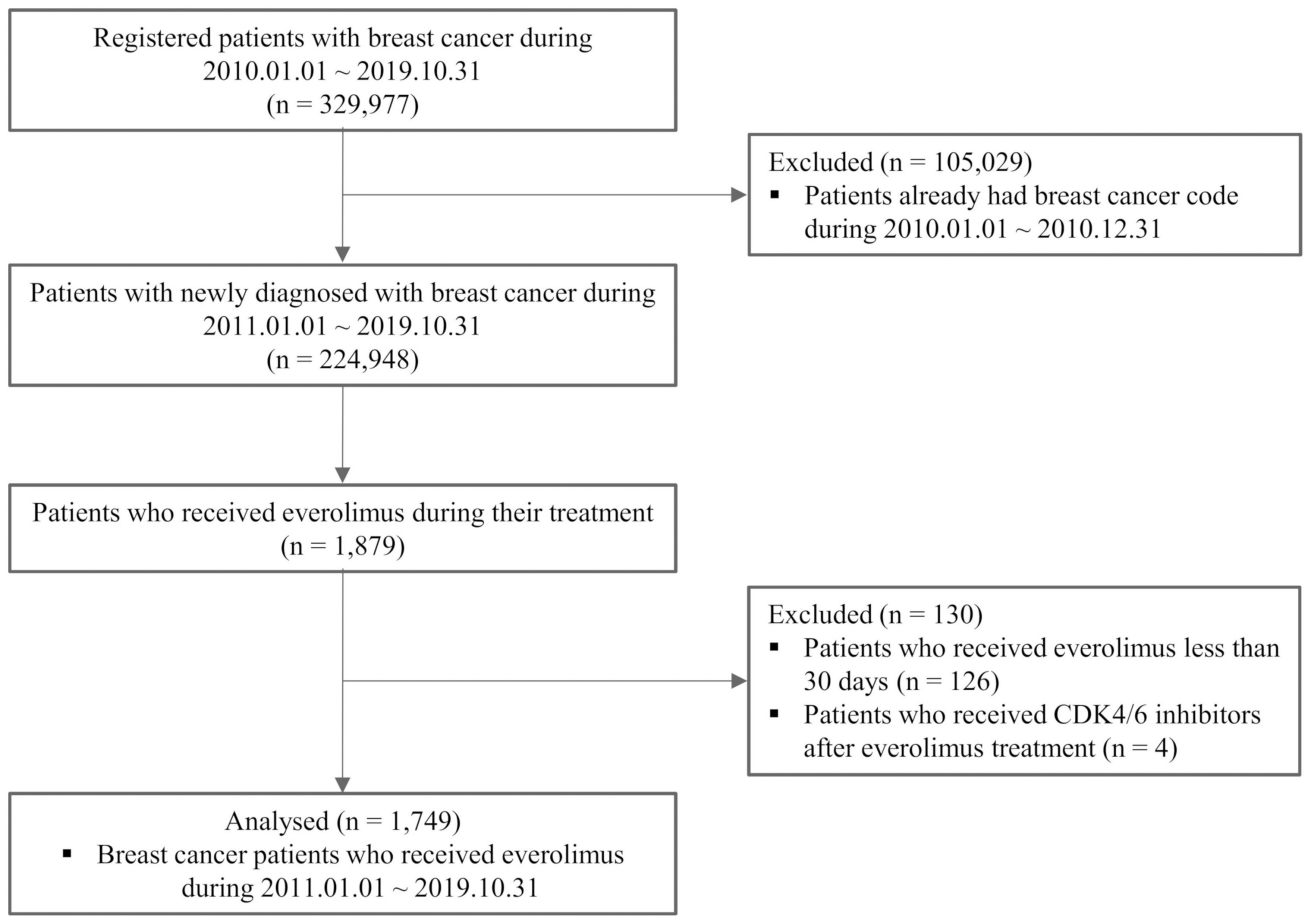
Summary of the patient flow diagram.

From January 2010 to October 2019, a total of 329,977 patients were registered under ICD‐10 codes for breast cancer in the Republic of Korea. Excluding patients who already had a breast cancer code in 2010, there were 224,948 newly diagnosed cases of breast cancer between January 2011 and October 2019. Among them, 1879 patients under everolimus with endocrine therapy (exemestane) were retrieved, and 1749 patients were finally enrolled in this study, excluding 126 patients who received EverX treatment for less than 30 days and four patients who received CDK4/6 inhibitors after EverX treatment (Figure 1).

Of a total of 1749 patients, 529 patients were identified being prescribed statins during EverX treatment. In order to explore the combined effect of statin and EverX, patients with a combined treatment period of 30 days or more were classified into the statin group, and 500 patients (28.6%) were included. Most of statins were lipophilic (*n* = 476, 95.2%), and the median combination period was 5.36 months (IQR, 2.63–11.27 months). Of note, 382 (76.4%) patients continued to take statin even after the end of EverX treatment, with median duration of 6.65 months (IQR, 2.10–17.91 months). Information on the total duration of statin use based on the EverX treatment period is summarized in Table S2. Compared with the no‐statin group (*n* = 1249, 71.4%), the median age of the patients was higher in the statin group (median age 61 vs. 55 years, *p* < 0.001), and the proportion of patients with diabetes (65.0% vs. 32.4%, *p* < 0.001), hypertension (62.2% vs. 25.3%, *p* < 0.001), and hyperlipidemia (88.4% vs. 41.2%, *p* < 0.001) was also significantly higher in the statin group. Approximately 95% of patients received an aromatase inhibitor (AI) prior to EverX, and the median duration of prior AI treatment was greater in the statin group (18.17 vs. 11.83 months, *p* < 0.001). Seventy‐one percent of all patients had received cytotoxic chemotherapy before EverX treatment, either in an adjuvant/neoadjuvant or metastatic setting. The proportion of patients receiving cytotoxic chemotherapy was slightly higher in the no‐statin group (72.5% vs. 67.8%, *p* = 0.05). Meanwhile, the number of cytotoxic chemotherapies after EverX treatment was not statistically different between the two groups. Baseline characteristics, including all patients' prior treatment history and comorbidities, are summarized in Table 1, and details of the chemotherapeutic agents that patients previously received are described in Table S3.

**TABLE 1:** Baseline characteristics of the study population

Variables	All patients (*n* = 1749)	No‐statin group (*n* = 1249)	Statin group (*n* = 500)	*p*
Age at starting EverX (yrs, median, IQR)	57 (51–64)	55 (50–63)	61 (55–68)	<0.001
≥ 60 yrs	724 (41.4%)	433 (34.7%)	291 (58.2%)	<0.001
< 60 yrs	1025 (58.6%)	816 (65.3%)	209 (41.8%)	
Prior AI treatment before EverX	1656 (94.7%)	1179 (94.4%)	477 (95.4%)	0.40
Duration of prior AI treatment (mon, median, range)	13.22 (5.80–26.25)	11.83 (5.29–23.82)	18.17 (8.41–30.49)	<0.001
> 1 yr	893 (53.9%)	581 (49.3%)	312 (65.4%)	<0.001
> 6 mon, ≤ 1 yr	322 (19.4%)	249 (21.1%)	73 (15.3%)	
≤ 6 mon	441 (26.6%)	349 (29.6%)	92 (19.3%)	
No. of cytotoxic chemotherapy before EverX	1244 (71.1%)	905 (72.5%)	339 (67.8%)	0.05
≤ 2	1128 (64.5%)	809 (63.3%)	319 (63.8%)	0.70
≥ 3	116 (6.6%)	96 (7.7%)	20 (4.0%)	0.005
No. of cytotoxic chemotherapy after EverX	971 (55.5%)	702 (56.2%)	269 (53.8%)	0.36
≤ 2	648 (37.0%)	469 (37.6%)	179 (35.8%)	0.49
≥ 3	323 (18.5%)	213 (17.1%)	90 (18.0%)	0.64
Co‐morbidity				
Hypertension	730 (41.7%)	405 (32.4%)	325 (65.0%)	<0.001
Diabetes mellitus (DM)	627 (35.8%)	316 (25.3%)	311 (62.2%)	<0.001
Existing DM	422 (67.3%)	219 (69.3%)	203 (65.3%)	0.28
De novo DM	205 (32.7%)	97 (30.7%)	105 (34.7%)	
Hyperlipidemia	956 (54.7%)	514 (41.2%)	442 (88.4%)	<0.001

Abbreviations: AI, aromatase inhibitor; EverX, everolimus and exemestane; IQR, interquartile range; mon, months; n, number; yr, year; yrs, years.

### 3.2 Survival analysis according to concomitant statin use

**FIGURE 2:**
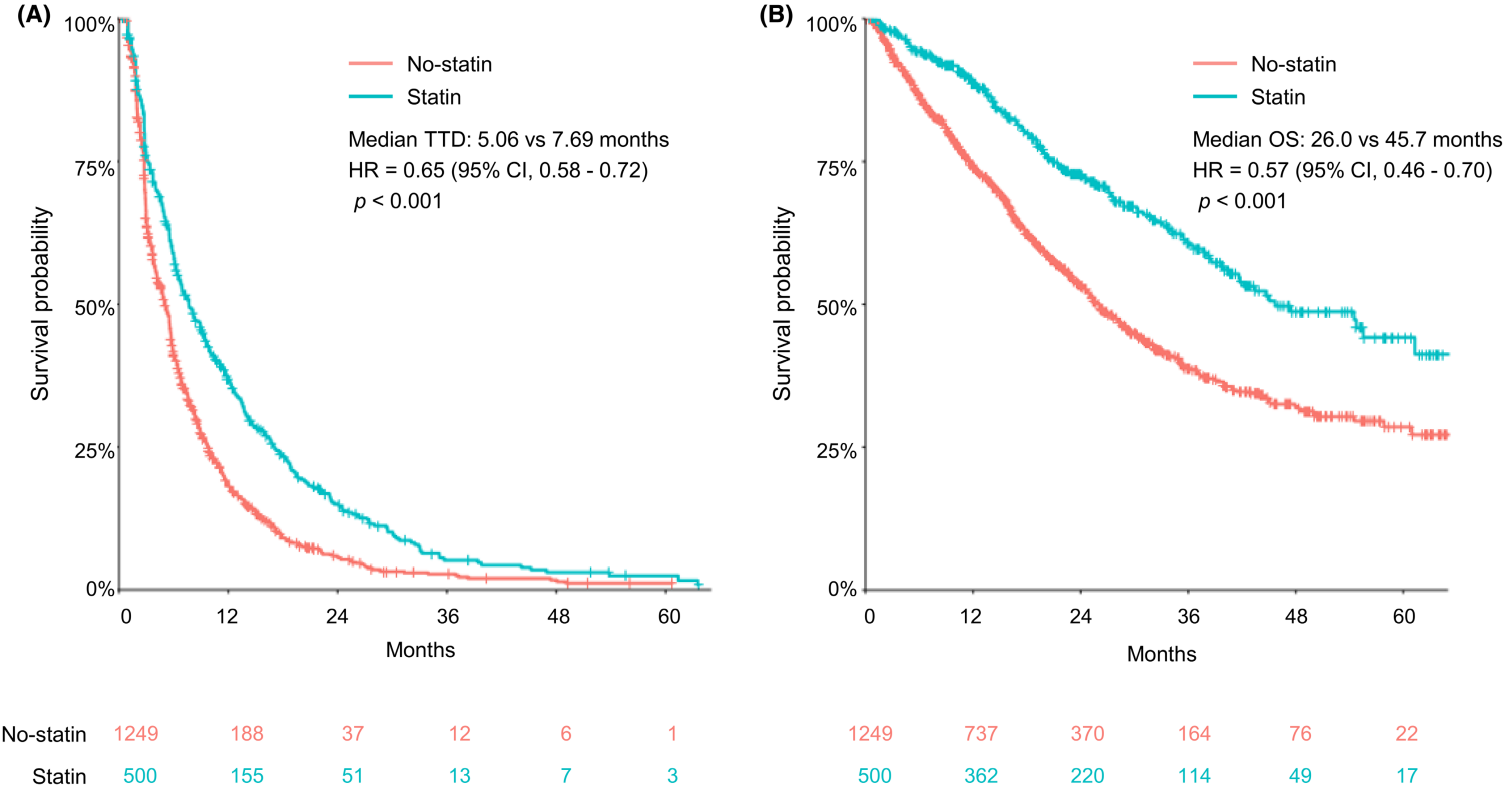
The Kaplan–Meier survival curves of the patients with and without exposure to statins during the EverX treatment. (A) TTD and (B) OS for EverX according to the concomitant use of statins in all patients.

**FIGURE 3:**
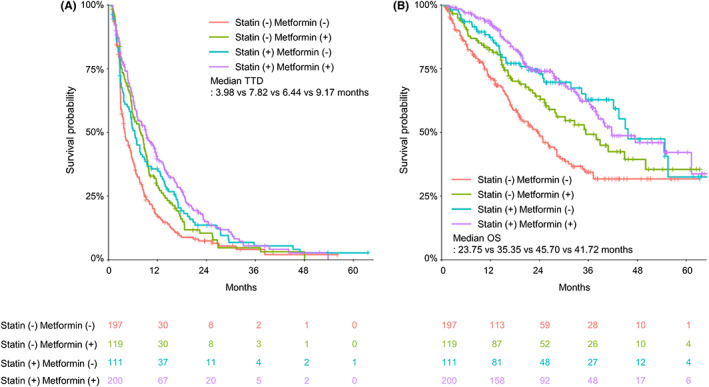
The Kaplan–Meier survival curves of the patients with and without exposure to statins and/or metformin during the EverX treatment in diabetic patients. (A) TTD and (B) OS for EverX according to the concomitant use of statin ± metformin.

In the HIRA database, only the prescribed duration of the drug was identified, and the reason for discontinuation was not known. Therefore, survival analysis using the TTD for EverX was performed to confirm the difference between the two groups. Both the median TTD and OS for EverX were significantly higher in the statin group than in the no‐statin group [7.69 vs. 5.06 months, hazard ratio (HR) = 0.65 (95% confidence interval [CI], 0.58–0.72), *p* < 0.001; 45.7 vs. 26.0 months, HR = 0.57 (95% CI, 0.46–0.70), *p* < 0.001, respectively] (Figure 2). Of the patients treated with EverX, 35.8% (*n* = 627) had diabetes and 49.6% (*n* = 311) of diabetic patients were taking statins. Metformin, a major treatment for diabetes, is known to have beneficial effects in cancer treatment. To address the interaction between metformin and statin treatment in diabetic patients, survival analyses were performed according to the use of statins and/or metformin. Patients who did not take statins or metformin had the shortest TTD and OS, while patients receiving both metformin and statin showed improved TTD and OS, indicating possible synergistic effects of the two drugs (Figure 3).

To differentiate between the effects of statins on their own and the synergistic effects of statins with EverX treatment, the no‐statin group was further divided into patients who had never been prescribed statins (never‐statin group, n = 988) and patients who had been prescribed statins before or after EverX treatment (ex‐statin group, n = 261). The median duration of statin use was 5.49 months (IQR, 1.87–25) in the ex‐statin group and most patients (n = 226, 86.6%) discontinued statins before EverX treatment. The median age of the ex‐statin group was similar to that of the statin group. On the other hand, the median duration of prior AI treatment was 14.65 months (range, 6.77–29.04), which showed a tendency to increase according to the duration of statin use when comparing the three groups (Table S4). Nevertheless, the median TTD and OS were significantly longer for the statin group, and that of the ex‐statin group did not differ from the never‐statin group (Figure S1).

### 3.3 Analyses of the benefit of concomitant statin use during EverX treatment

In the univariable Cox proportional hazard analysis, factors significantly associated with a shorter TTD for EverX included prior AI treatment duration (≤6 months) and a history of prior cytotoxic chemotherapy. On the other hand, comorbidity with DM or hyperlipidemia, metformin use, and statin use were identified as favorable factors for a longer TTD for EverX. Multivariable analysis showed that prior AI treatment duration (≤6 months) and a history of prior cytotoxic chemotherapy were independent factors associated with a shorter TTD for EverX, while younger age (< 60 years), metformin use, and statin use were independently associated with a longer TTD for EverX. The same results were found in the analyses of OS (Table 2). When additional Cox proportional hazard analysis for TTD for EverX was performed in diabetic patients only (*n* = 627), the use of statins, along with the use of metformin and de novo DM, remained an independent favorable factor for EverX treatment in this population (Table S5).

**TABLE 2:** Cox proportional hazard analysis for TTD and OS of EverX in the overall study population

	TTD						OS					
Covariates	Univariable			Multivariable			Univariable			Multivariable		
	HR	95% CI	*p*	HR	95% CI	*p*	HR	95% CI	*p*	HR	95% CI	*p*
Age at starting EverX												
≥ 60 yrs	Ref			Ref			Ref			Ref		
< 60 yrs	0.944	0.852–1.045	0.267	0.835	0.746–0.936	0.002	1.045	0.903–1.211	0.553	0.787	0.667–0.929	0.005
Duration of prior AI treatment												
> 1 yr	Ref			Ref			Ref			Ref		
> 6 mon, ≤ 1 yr	1.191	1.041–1.363	0.011	1.129	0.985–1.294	0.081	1.543	1.277–1.864	<0.001	1.393	1.149–1.689	0.001
≤ 6 mon	1.221	1.080–1.381	0.001	1.170	1.030–1.328	0.016	1.666	1.404–1.977	<0.001	1.503	1.257–1.796	<0.001
Prior cytotoxic chemotherapy												
No	Ref			Ref			Ref			Ref		
Yes	1.319	1.175–1.480	<0.001	1.262	1.117–1.425	<0.001	1.774	1.508–2.088	<0.001	1.56	1.313–1.854	<0.001
Hypertension												
No	Ref			Ref			Ref			Ref		
Yes	0.947	0.854–1.049	0.293	1.064	0.947–1.195	0.298	0.834	0.720–0.967	0.016	1.016	0.859–1.202	0.856
Diabetes mellitus												
No	Ref			Ref			Ref			Ref		
Yes	0.787	0.708–0.875	<0.001	0.997	0.866–1.147	0.965	0.784	0.673–0.913	0.002	1.102	0.903–1.346	0.339
Hyperlipidemia												
No	Ref			Ref			Ref			Ref		
Yes	0.847	0.765–0.937	0.001	0.976	0.868–1.098	0.688	0.722	0.625–0.833	<0.001	0.873	0.741–1.029	0.104
Use of statin												
No	Ref			Ref			Ref			Ref		
Yes	0.646	0.576–0.724	<0.001	0.673	0.588–0.771	<0.001	0.533	0.448–0.636	<0.001	0.572	0.464–0.704	<0.001
Use of metformin												
No	Ref			Ref			Ref			Ref		
Yes	0.68	0.596–0.774	<0.001	0.764	0.645–0.905	0.002	0.651	0.535–0.793	<0.001	0.757	0.587–0.976	0.032

Abbreviations: AI, aromatase inhibitor; CI, confidence interval; HR, hazard ratio; EverX, everolimus and exemestane; OS, overall survival; mon, months; Ref, reference; TTD, time to treatment duration; yr, year; yrs, years.

**FIGURE 4:**
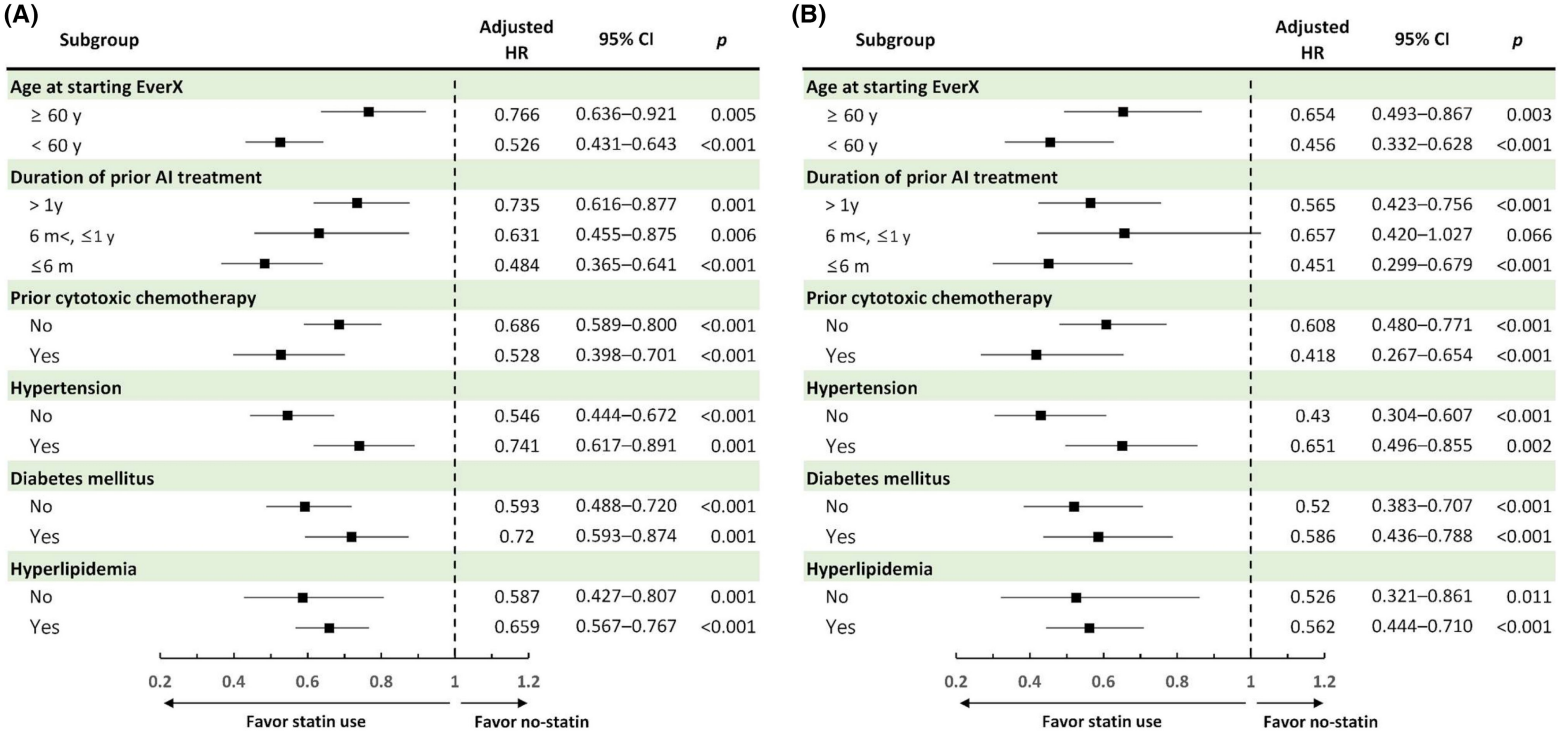
Forest plot of the benefit of concomitant statins on (A) TTF and (B) OS for EverX treatment in patient subgroups. Adjusted variables: age at the start of EverX treatment (≥ 60 or < 60 years), duration of prior AI treatment, prior cytotoxic chemotherapy, and comorbidities including hypertension, diabetes mellitus, and hyperlipidemia. HR, hazard ratio; CI, confidence interval.

Subgroup analyses were further performed to determine whether the beneficial effects of statins were consistent across the different baseline characteristics of patients. The favorable effects of statins were demonstrated consistently across all subgroups of patients in both TTD and OS for EverX treatment (Figure 4).

## 4 DISCUSSION

Our study showed an association between concomitant statin use and favorable survival outcomes in HR+ ABC patients treated with an EverX regimen. In this study, the patients who received statins concomitantly with EverX showed significantly longer TTD and OS compared to the rest of the patients who did not. The beneficial effect of statins was confirmed to be an independent factor even after adjustment for other clinical factors and the effect was demonstrated consistently across all subgroups of patients. In particular, it is widely accepted that metformin has anticancer effects through several studies. Considering that metformin is the most common diabetic medication and many diabetic patients are being treated with hyperlipidemia, we included metformin as one of the factors in analyses and showed the independent effect of statins regardless of metformin.

Several studies have suggested the beneficial effects of statin use in patients with breast cancer.^17^, ^18^, ^19^ Previous studies have indicated an association between the use of statins and a reduced risk of breast cancer recurrence,^20^ and long‐term post‐diagnostic statin treatment has been associated with a reduced risk of contralateral breast cancer.^21^ Among patients with HR+ breast cancer, statins have been shown to have a favorable impact on breast cancer recurrence and mortality when combined with adjuvant endocrine treatment.^18^, ^20^, ^22^ A recent Danish study performed in a modern cohort of AI‐treated early breast cancer patients also provided a consecutive association between statin use and a reduction in breast cancer recurrence.^23^ While all these clinical studies showed the beneficial effects of statins in early breast cancer patients, most studies showing the anti‐cancer effects of statins in ABC were limited to preclinical experiments. To the best of our knowledge, this is the first study to demonstrate the beneficial effects of statins in the treatment of HR+ ABC using real‐world clinical data.

Since their approval in the late 1980 s, statins have revolutionized the clinical management of hypercholesterolemia.^7^, ^24^ Cholesterol is essential for membrane synthesis in dividing cells, and highly proliferative cells such as cancer cells require increased cholesterol biosynthesis.^25^ Cholesterol has also been known to play a variety of roles in tumorigenesis,^26^, ^27^ and in this specific context, statins have been suggested to have anticancer effects through the depletion of cholesterol.^24^ Furthermore, statin treatment reduced the levels of 27HC in tumor cells. 27HC potentiates ER‐dependent tumor growth by acting as an endogenous ER modulator as well as a liver X receptor agonist,^4^, ^5^, ^28^ and it promotes metastasis through interactions with myeloid immune cells.^29^ Recent studies have shown that chronic estrogen deprivation in ER‐positive breast cancer cells resulted in epigenetic activation of the mevalonate pathway and accumulation of other ligands, including 27HC, which potentiates ER signaling even in the absence of estrogen.^30^, ^31^

In this study, 77.6% of patients (*n* = 388) in the statin group had already taken statins for a significant period prior to EverX treatment. It seems that long‐term use of statins not only enhances the clinical effects of endocrine therapy, but also have a positive effect on overall survival. The rationale for this assumption is that there were more patients in the statin group who showed a response to prior endocrine therapy for more than 1 year (no statin group vs. statin group, 49.3% vs. 65.4%, *p* < 0.001). A longer duration of prior AI treatment was also found in the ex‐statin group compared to the never‐statin group. However, most patients discontinued statin prior to EverX treatment, which may be the reason why the TTD and OS of the ex‐statin group were rather similar to those in the never‐statin group. Of note, given the anonymized data lacking detailed clinical information of each patients and retrospective nature of this study, we could only surmise the favorable survival impact of statins.

Multiple in vitro and in vivo studies have demonstrated that activation of the PI3K/AKT/mTOR pathway induces resistance to endocrine treatment^32^, ^33^ and the mTOR inhibitor everolimus showed clinical efficacy with exemestane in HR+ ABC by targeting this pathway.^34^ One of the mechanisms of everolimus resistance is that inhibition of mTORC1 leads to overactivation of the PI3K/AKT and RAS/RAF/MEK/MAPK pathways as the negative feedback loop disappears.^35^, ^36^ Activation of the mevalonate pathway is known to induce prenylation of small GTPases such as Rho, Ras, or Rab, which leads to activation of the PI3K‐AKT–mTOR and RAS–MEK–ERK pathways.^37^ Based on these findings, statins may have synergistic effects with everolimus by inhibiting the mevalonate pathway, which has already been noted in several in vitro studies.^38^, ^39^ In real clinical practice, we do not use everolimus alone, but in combination with exemestane in the treatment of patients with HR+ ABC. Therefore, we could not distinguish the two drugs from each other, and we analyzed them in one regimen, EverX. Our findings that the TTD for EverX was significantly increased with a statin combination was a clinical demonstration of a possible synergistic effect between statins and everolimus with endocrine therapy. Further research should be undertaken to clarify the exact mechanism of statins' effects in HR+ ABC.

The main weakness of this study was the paucity of detailed clinical information about the patients. As the HIRA data constituted the medical claim data, we were not able to determine the disease extent (with or without visceral metastases) of each patient, the exact reason for treatment discontinuation (disease progression or adverse effects), and the reason for death (cancer‐related or not). Furthermore, because of the lack of laboratory data, we could not evaluate the difference in cholesterol levels between the two groups, the reasons for using statins, or the amount by which cholesterol levels fell in individual patients after statin treatment. In addition, various types of statins were not distinguished in this study. It has been hypothesized that lipophilic statins are more likely to reach and easily enter extrahepatic cells, and previous epidemiologic studies have shown that lipophilic, but not hydrophilic, statin use is associated with reduced breast cancer incidence and recurrence.^20^, ^24^, ^40^ Nevertheless, the strength of this study is that the clinical usefulness of long‐term use of statins and their concomitant use with EverX in patients with advanced HR+ breast cancer, which is difficult to evaluate in clinical trials, was confirmed with large‐scale medical data collected from real‐world patients.

## 5 CONCLUSIONS

Taken together, our results indicate that statins, commonly used to lower systemic cholesterol levels, could be associated with favorable survival outcomes in the treatment of patients with HR+ ABC. Statins may have synergistic effects with endocrine therapy with the mTOR inhibitor everolimus and may improve the treatment outcomes of EverX. Further research is needed to validate these findings and to explore the appropriate use of statins in the treatment of breast cancer.

## AUTHOR CONTRIBUTIONS

**Kyoungmin Lee:** Formal analysis (equal); funding acquisition (equal); methodology (equal); validation (equal); writing – original draft (equal). **Eunjin Noh:** Formal analysis (equal); investigation (equal); methodology (equal); software (equal). **Seok Joo Moon:** Formal analysis (equal); investigation (equal); methodology (equal); software (equal). **Yoonjung Yoonie Joo:** Formal analysis (equal); investigation (equal); methodology (equal). **Eun Joo Kang:** Conceptualization (equal); resources (equal); validation (equal). **Jae Hong Seo:** Resources (equal); supervision (equal); validation (equal). **In Hae Park:** Conceptualization (equal); funding acquisition (equal); methodology (equal); project administration (equal); writing – review and editing (equal).

## FUNDING INFORMATION

This work was supported by Korea University Guro Hospital (KOREA RESEARCH‐DRIVEN HOSPITAL) and by grants funded by Korea University Medicine (No. K2108041 to KL; No. K2023211 to IHP).

## CONFLICT OF INTEREST

There are no conflicts of interest relevant to this article to report.

## ETHICS STATEMENT

This study was approved by the Institutional Review Board of the Korea University Guro Hospital (IRB approval number: 2020GR0282), and performed in accordance with the Declaration of Helsinki. Written informed consent was waived due to the nature of the study.

## Supporting information


Figure S1.

Table S1.

Table S2.

Table S3.

Table S4.

Table S5.

## Data Availability

The original contributions presented in the study are included in the article/supplementary material, further inquiries can be directed to the corresponding authors.
