# Supplementary material for: Statin use in patients with hormone receptor‐positive metastatic breast cancer treated with everolimus and exemestane
Source: Cancer Med. 2022 Oct 19;12(5):5461–70. doi: 10.1002/cam4.5369 (PMC10028110; doi:10.1002/cam4.5369)
Supplement: Supplementary file 1 — Figure S1. [file CAM4-12-5461-s006.pdf]

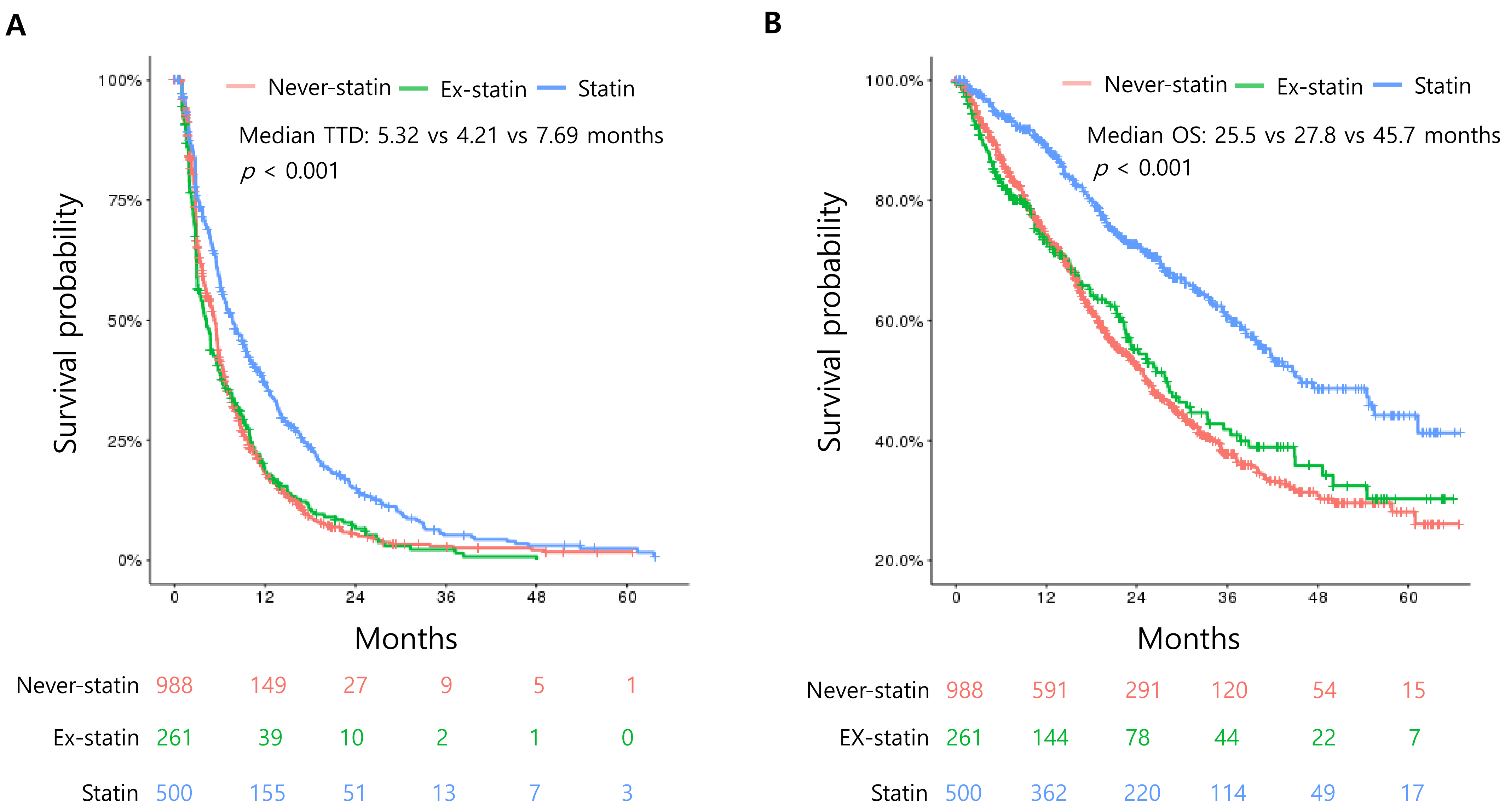

Figure S1. The Kaplan-Meier survival curves between the three groups of patients (never-statin group vs ex-statin group vs statin group).
(A) TTD and (B) OS for EverX according to the concomitant use of statins in all patients.
