# Supplementary material for: Statin use in patients with hormone receptor‐positive metastatic breast cancer treated with everolimus and exemestane
Source: Cancer Med. 2022 Oct 19;12(5):5461–70. doi: 10.1002/cam4.5369 (PMC10028110; doi:10.1002/cam4.5369)
Supplement: Supplementary file 2 — Table S1. [file CAM4-12-5461-s003.docx]

**Table S1. Ingredient codes used in this study**

| Ingredient code | MFDS drug class | Ingredient | Dosage | Administration route |  |
| --- | --- | --- | --- | --- | --- |
| *<Statin>* | | | | |  |
| 111501ATB | 218 | atorvastatin calcium | as atorvastatin 10mg | Oral |  |
| 111502ATB | 218 | atorvastatin calcium | as atorvastatin 20mg | Oral |  |
| 111503ATB | 218 | atorvastatin calcium | as atorvastatin 40mg | Oral |  |
| 111504ATB | 218 | atorvastatin calcium | as atorvastatin 80mg | Oral |  |
| 162401ACH | 218 | fluvastatin | 20mg | Oral |  |
| 162402ACH | 218 | fluvastatin | 40mg | Oral |  |
| 162403ATR | 218 | fluvastatin | 80mg | Oral |  |
| 185801ATB | 218 | lovastatin | 20mg | Oral |  |
| 216601ATB | 218 | pravastatin sodium | 10mg | Oral |  |
| 216602ATB | 218 | pravastatin sodium | 5mg | Oral |  |
| 216603ATB | 218 | pravastatin sodium | 20mg | Oral |  |
| 216604ATB | 218 | pravastatin sodium | 40mg | Oral |  |
| 227801ATB | 218 | simvastatin | 20mg | Oral |  |
| 227801ATR | 218 | simvastatin | 20mg | Oral |  |
| 227802ATB | 218 | simvastatin | 40mg | Oral |  |
| 227806ATB | 218 | simvastatin | 5mg | Oral |  |
| 454001ATB | 218 | rosuvastatin calcium | as rosuvastatin 10mg | Oral |  |
| 454002ATB | 218 | rosuvastatin calcium | as rosuvastatin 20mg | Oral |  |
| 454003ATB | 218 | rosuvastatin calcium | as rosuvastatin 5mg | Oral |  |
| 470901ATB | 218 | pitavastatin calcium | 2mg | Oral |  |
| 470902ATB | 218 | pitavastatin calcium | 1mg | Oral |  |
| 470903ATB | 218 | pitavastatin calcium | 4mg | Oral |  |
| 471000ATB | 218 | simvastatin | 10mg | Oral |  |
| 471100ATB | 218 | simvastatin | 20mg | Oral |  |
| 507800ATB | 218 | simvastatin | 40mg | Oral |  |
| 502201ATB | 218 | atorvastatin strontium pentahydrate | as atorvastatin 10mg | Oral |  |
| 502202ATB | 218 | atorvastatin strontium pentahydrate | as atorvastatin 20mg | Oral |  |
| 502203ATB | 218 | atorvastatin strontium pentahydrate | as atorvastatin 40mg | Oral |  |
| 502204ATB | 218 | atorvastatin strontium pentahydrate | as atorvastatin 80mg | Oral |  |
| 519300ACH | 218 | pravastatin sodium | 40mg | Oral |  |
| 631400ATB | 218 | simvastatin | 20mg | Oral |  |
| 631500ATB | 218 | simvastatin | 40mg | Oral |  |
| 633800ATB | 218 | atorvastatin calcium | as atorvastatin 10mg | Oral |  |
| 633900ATB | 218 | atorvastatin calcium | as atorvastatin 20mg | Oral |  |
| 634600ATB | 218 | atorvastatin calcium | as atorvastatin 80mg | Oral |  |
| 634800ATB | 218 | atorvastatin calcium | as atorvastatin 40mg | Oral |  |
| 640700ATB | 218 | rosuvastatin calcium | as rosuvastatin 5mg | Oral |  |
| 640800ATB | 218 | rosuvastatin calcium | as rosuvastatin 10mg | Oral |  |
| 640900ATB | 218 | rosuvastatin calcium | as rosuvastatin 20mg | Oral |  |
| 663400ACS | 218 | rosuvastatin calcium | as rosuvastatin 5mg | Oral |  |
| 679300ACH | 218 | pitavastatin calcium | 2mg | Oral |  |
| 472300ATB | 219 | atorvastatin calcium | as atorvastatin 10mg | Oral |  |
| 472400ATB | 219 | atorvastatin calcium | as atorvastatin 20mg | Oral |  |
| 472500ATB | 219 | atorvastatin calcium | as atorvastatin 40mg | Oral |  |
| 518900ATB | 219 | atorvastatin calcium | as atorvastatin 20mg | Oral |  |
| 524000ATB | 219 | atorvastatin calcium | as atorvastatin 10mg | Oral |  |
| 524100ATB | 219 | atorvastatin calcium | as atorvastatin 20mg | Oral |  |
| 527000ATB | 219 | atorvastatin calcium | as atorvastatin 20mg | Oral |  |
| 527100ATB | 219 | atorvastatin calcium | as atorvastatin 10mg | Oral |  |
| 525000ATB | 219 | rosuvastatin calcium | as rosuvastatin 10mg | Oral |  |
| 525100ATB | 219 | rosuvastatin calcium | as rosuvastatin 10mg | Oral |  |
| 525200ATB | 219 | rosuvastatin calcium | as rosuvastatin 20mg | Oral |  |
| 525300ATB | 219 | rosuvastatin calcium | as rosuvastatin 20mg | Oral |  |
| 629700ATB | 219 | rosuvastatin calcium | as rosuvastatin 5mg | Oral |  |
| 629800ATB | 219 | rosuvastatin calcium | as rosuvastatin 5mg | Oral |  |
| 526300ATB | 219 | rosuvastatin calcium | as rosuvastatin 10mg | Oral |  |
| 526400ATB | 219 | rosuvastatin calcium | as rosuvastatin 20mg | Oral |  |
| 526500ATB | 219 | rosuvastatin calcium | as rosuvastatin 20mg | Oral |  |
| 526900ATB | 219 | rosuvastatin calcium | as rosuvastatin 5mg | Oral |  |
| 644100ATB | 219 | rosuvastatin calcium | as rosuvastatin 10mg | Oral |  |
| 644200ATB | 219 | rosuvastatin calcium | as rosuvastatin 5mg | Oral |  |
| 653200ATB | 219 | rosuvastatin calcium | as rosuvastatin 10mg | Oral |  |
| 614500ATB | 219 | atorvastatin calcium | as atorvastatin 10mg | Oral |  |
| 629900ATB | 219 | rosuvastatin calcium | as rosuvastatin 10mg | Oral |  |
| 630000ATB | 219 | rosuvastatin calcium | as rosuvastatin 20mg | Oral |  |
| 630100ATB | 219 | rosuvastatin calcium | as rosuvastatin 10mg | Oral |  |
| 630200ATB | 219 | rosuvastatin calcium | as rosuvastatin 20mg | Oral |  |
| 631600ATB | 219 | rosuvastatin calcium | as rosuvastatin 5mg | Oral |  |
| 631700ATB | 219 | rosuvastatin calcium | as rosuvastatin 5mg | Oral |  |
| 634900ATB | 219 | pitavastatin calcium | 2mg | Oral |  |
| 635000ATB | 219 | pitavastatin calcium | 2mg | Oral |  |
| 635100ATB | 219 | pitavastatin calcium | 4mg | Oral |  |
| 635200ATB | 219 | pitavastatin calcium | 4mg | Oral |  |
| 654700ATB | 219 | rosuvastatin calcium | as rosuvastatin 10mg | Oral |  |
| 654800ATB | 219 | rosuvastatin calcium | as rosuvastatin 5mg | Oral |  |
| 654900ATB | 219 | rosuvastatin calcium | as rosuvastatin 10mg | Oral |  |
| 655000ATB | 219 | rosuvastatin calcium | as rosuvastatin 5mg | Oral |  |
| 661800ATB | 219 | rosuvastatin calcium | as rosuvastatin 5mg | Oral |  |
| 661900ATB | 219 | rosuvastatin calcium | as rosuvastatin 10mg | Oral |  |
| 662000ATB | 219 | rosuvastatin calcium | as rosuvastatin 10mg | Oral |  |
| 662100ATB | 219 | rosuvastatin calcium | as rosuvastatin 20mg | Oral |  |
| 673700ATB | 219 | rosuvastatin calcium | as rosuvastatin 5mg | Oral |  |
| 663900ATB | 219 | rosuvastatin calcium | as rosuvastatin 5mg | Oral |  |
| 664000ATB | 219 | rosuvastatin calcium | as rosuvastatin 10mg | Oral |  |
| 664100ATB | 219 | rosuvastatin calcium | as rosuvastatin 20mg | Oral |  |
| 664200ATB | 219 | rosuvastatin calcium | as rosuvastatin 5mg | Oral |  |
| 664300ATB | 219 | rosuvastatin calcium | as rosuvastatin 10mg | Oral |  |
| 664400ATB | 219 | rosuvastatin calcium | as rosuvastatin 20mg | Oral |  |
| 671200ATB | 219 | rosuvastatin calcium | as rosuvastatin 5mg | Oral |  |
| 671300ATB | 219 | rosuvastatin calcium | as rosuvastatin 5mg | Oral |  |
| 671400ATB | 219 | rosuvastatin calcium | as rosuvastatin 10mg | Oral |  |
| 671500ATB | 219 | rosuvastatin calcium | as rosuvastatin 10mg | Oral |  |
| 671600ATB | 219 | rosuvastatin calcium | as rosuvastatin 10mg | Oral |  |
| 671700ATB | 219 | rosuvastatin calcium | as rosuvastatin 20mg | Oral |  |
| 677000ATB | 219 | rosuvastatin calcium | as rosuvastatin 20mg | Oral |  |
| 677100ATB | 219 | rosuvastatin calcium | as rosuvastatin 20mg | Oral |  |
| 673900ATB | 219 | rosuvastatin calcium | as rosuvastatin 5mg | Oral |  |
| 674000ATB | 219 | rosuvastatin calcium | as rosuvastatin 10mg | Oral |  |
| 674100ATB | 219 | rosuvastatin calcium | as rosuvastatin 20mg | Oral |  |
| 678600ATB | 219 | rosuvastatin calcium | as rosuvastatin 10mg | Oral |  |
| 677300ATB | 219 | rosuvastatin calcium | as rosuvastatin 5mg | Oral |  |
| 677400ATB | 219 | rosuvastatin calcium | as rosuvastatin 10mg | Oral |  |
| 677500ATB | 219 | rosuvastatin calcium | as rosuvastatin 10mg | Oral |  |
| 677600ATB | 219 | rosuvastatin calcium | as rosuvastatin 20mg | Oral |  |
| 686800ATB | 219 | rosuvastatin calcium | as rosuvastatin 5mg | Oral |  |
| 686900ATB | 219 | rosuvastatin calcium | as rosuvastatin 10mg | Oral |  |
| 679500ATB | 219 | rosuvastatin calcium | as rosuvastatin 10mg | Oral |  |
| 679600ATB | 219 | rosuvastatin calcium | as rosuvastatin 10mg | Oral |  |
| 679700ATB | 219 | rosuvastatin calcium | as rosuvastatin 5mg | Oral |  |
| 680300ATB | 219 | rosuvastatin calcium | as rosuvastatin 5mg | Oral |  |
| 683000ATB | 219 | rosuvastatin calcium | as rosuvastatin 10mg | Oral |  |
| 683100ATB | 219 | rosuvastatin calcium | as rosuvastatin 20mg | Oral |  |
| 683200ATB | 219 | rosuvastatin calcium | as rosuvastatin 10mg | Oral |  |
| 684300ATB | 219 | rosuvastatin calcium | as rosuvastatin 5mg | Oral |  |
| 684400ATB | 219 | rosuvastatin calcium | as rosuvastatin 10mg | Oral |  |
| 684500ATB | 219 | rosuvastatin calcium | as rosuvastatin 5mg | Oral |  |
| 684600ATB | 219 | rosuvastatin calcium | as rosuvastatin 10mg | Oral |  |
| 684700ATB | 219 | rosuvastatin calcium | as rosuvastatin 20mg | Oral |  |
| 688100ATB | 219 | atorvastatin calcium | as atorvastatin 10mg | Oral |  |
| 688200ATB | 219 | atorvastatin calcium | as atorvastatin 10mg | Oral |  |
| 688300ATB | 219 | atorvastatin calcium | as atorvastatin 20mg | Oral |  |
| 688400ATB | 219 | atorvastatin calcium | as atorvastatin 20mg | Oral |  |
| 688500ATB | 219 | atorvastatin calcium | as atorvastatin 40mg | Oral |  |
| 664600ATB | 396 | rosuvastatin calcium | as rosuvastatin 5mg | Oral |  |
| 664700ATB | 396 | rosuvastatin calcium | as rosuvastatin 10mg | Oral |  |
| 664800ATB | 396 | rosuvastatin calcium | as rosuvastatin 20mg | Oral |  |
| 671800ATR | 396 | atorvastatin calcium | as atorvastatin 10mg | Oral |  |
| 673800ATR | 396 | atorvastatin calcium | as atorvastatin 10mg | Oral |  |
| 671900ATR | 396 | atorvastatin calcium | as atorvastatin 10mg | Oral |  |
| 672000ATR | 396 | atorvastatin calcium | as atorvastatin 20mg | Oral |  |
| 672100ATR | 396 | atorvastatin calcium | as atorvastatin 20mg | Oral |  |
| 672500ATR | 396 | rosuvastatin calcium | as rosuvastatin 5mg | Oral |  |
| 672600ATR | 396 | rosuvastatin calcium | as rosuvastatin 5mg | Oral |  |
| 672800ATR | 396 | rosuvastatin calcium | as rosuvastatin 10mg | Oral |  |
| 672900ATR | 396 | rosuvastatin calcium | as rosuvastatin 10mg | Oral |  |
| 673000ATR | 396 | rosuvastatin calcium | as rosuvastatin 20mg | Oral |  |
| 683300ATR | 396 | rosuvastatin calcium | as rosuvastatin 5mg | Oral |  |
| 683400ATR | 396 | rosuvastatin calcium | as rosuvastatin 10mg | Oral |  |
| *<Metformin>* | | | | | |
| 191501ATB | 396 | metformin hydrochloride | 0.25g | Oral |  |
| 191502ATB | 396 | metformin hydrochloride | 0.5g | Oral |  |
| 191502ATR | 396 | metformin hydrochloride | 0.5g | Oral |  |
| 191503ATB | 396 | metformin hydrochloride | 0.85g | Oral |  |
| 191504ATB | 396 | metformin hydrochloride | 1g | Oral |  |
| 191504ATR | 396 | metformin hydrochloride | 1g | Oral |  |
| 191505ATR | 396 | metformin hydrochloride | 0.75g | Oral |  |
| 443400ATB | 396 | metformin hydrochloride | 0.5g | Oral |  |
| 443500ATB | 396 | metformin hydrochloride | 0.5g | Oral |  |
| 474200ATB | 396 | metformin hydrochloride | 0.25g | Oral |  |
| 474300ATB | 396 | metformin hydrochloride | 0.5g | Oral |  |
| 474300ATR | 396 | metformin hydrochloride | 0.5g | Oral |  |
| 498600ATB | 396 | metformin hydrochloride | 0.5g | Oral |  |
| 497200ATB | 396 | metformin hydrochloride | 0.5g | Oral |  |
| 498100ATB | 396 | metformin hydrochloride | 0.85g | Oral |  |
| 502300ATB | 396 | metformin hydrochloride | 1g | Oral |  |
| 502300ATR | 396 | metformin hydrochloride | 1g | Oral |  |
| 502900ATB | 396 | metformin hydrochloride | 0.85g | Oral |  |
| 513700ATB | 396 | metformin hydrochloride | 0.5g | Oral |  |
| 513700ATR | 396 | metformin hydrochloride | 0.5g | Oral |  |
| 524700ATR | 396 | metformin hydrochloride | 1g | Oral |  |
| 507000ATB | 396 | metformin hydrochloride | 0.85g | Oral |  |
| 507100ATB | 396 | metformin hydrochloride | 1g | Oral |  |
| 519600ATB | 396 | metformin hydrochloride | 0.5g | Oral |  |
| 518500ATR | 396 | metformin hydrochloride | 0.5g | Oral |  |
| 518600ATR | 396 | metformin hydrochloride | 1g | Oral |  |
| 518800ATB | 396 | metformin hydrochloride | 0.5g | Oral |  |
| 520500ATB | 396 | metformin hydrochloride | 1g | Oral |  |
| 520600ATB | 396 | metformin hydrochloride | 0.85g | Oral |  |
| 520700ATB | 396 | metformin hydrochloride | 0.25g | Oral |  |
| 523600ATB | 396 | metformin hydrochloride | 0.5g | Oral |  |
| 523700ATB | 396 | metformin hydrochloride | 0.5g | Oral |  |
| 523800ATR | 396 | metformin hydrochloride | 0.5g | Oral |  |
| 632000ATR | 396 | metformin hydrochloride | 1g | Oral |  |
| 645000ATR | 396 | metformin hydrochloride | 0.5g | Oral |  |
| 654100ATR | 396 | metformin hydrochloride | 1g | Oral |  |
| 635600ATB | 396 | metformin hydrochloride | 0.5g | Oral |  |
| 635700ATB | 396 | metformin hydrochloride | 1g | Oral |  |
| 675500ATB | 396 | metformin hydrochloride | 0.85g | Oral |  |
| 639800ATR | 396 | metformin hydrochloride | 1g | Oral |  |
| 641400ATR | 396 | metformin hydrochloride | 0.5g | Oral |  |
| 641800ATR | 396 | metformin hydrochloride | 0.5g | Oral |  |
| 641900ATR | 396 | metformin hydrochloride | 0.75mg | Oral |  |
| 642000ATR | 396 | metformin hydrochloride | 1g | Oral |  |
| 648400ATB | 396 | metformin hydrochloride | 1g | Oral |  |
| 648500ATB | 396 | metformin hydrochloride | 0.5g | Oral |  |
| 648600ATB | 396 | metformin hydrochloride | 0.85g | Oral |  |
| 649000ATB | 396 | metformin hydrochloride | 1g | Oral |  |
| 649100ATB | 396 | metformin hydrochloride | 0.85g | Oral |  |
| 649200ATB | 396 | metformin hydrochloride | 1g | Oral |  |
| 649300ATB | 396 | metformin hydrochloride | 0.5g | Oral |  |
| 649400ATB | 396 | metformin hydrochloride | 0.5g | Oral |  |
| 649500ATB | 396 | metformin hydrochloride | 0.85g | Oral |  |
| 649900ATR | 396 | metformin hydrochloride | 1g | Oral |  |
| 650000ATR | 396 | metformin hydrochloride | 0.85g | Oral |  |
| 650100ATR | 396 | metformin hydrochloride | 0.5g | Oral |  |
| 653800ATR | 396 | metformin hydrochloride | 0.75g | Oral |  |
| 653900ATR | 396 | metformin hydrochloride | 1g | Oral |  |
| 654000ATR | 396 | metformin hydrochloride | 1g | Oral |  |
| 655700ATR | 396 | metformin hydrochloride | 0.5g | Oral |  |
| 671800ATR | 396 | metformin hydrochloride | 0.5g | Oral |  |
| 673800ATR | 396 | metformin hydrochloride | 1g | Oral |  |
| 671900ATR | 396 | metformin hydrochloride | 0.75g | Oral |  |
| 672000ATR | 396 | metformin hydrochloride | 0.5g | Oral |  |
| 672100ATR | 396 | metformin hydrochloride | 0.75g | Oral |  |
| 672500ATR | 396 | metformin hydrochloride | 0.5g | Oral |  |
| 672600ATR | 396 | metformin hydrochloride | 0.75g | Oral |  |
| 672700ATR | 396 | metformin hydrochloride | 0.5g | Oral |  |
| 672800ATR | 396 | metformin hydrochloride | 0.75g | Oral |  |
| 672900ATR | 396 | metformin hydrochloride | 0.5g | Oral |  |
| 673000ATR | 396 | metformin hydrochloride | 0.75g | Oral |  |
| 683300ATR | 396 | metformin hydrochloride | 1g | Oral |  |
| 683400ATR | 396 | metformin hydrochloride | 1g | Oral |  |
| *<Insulin>* | | | | |  |
| 170130BIJ | 396 | human insulin | 300I.U (100I.U/mL) | Parenteral |  |
| 170430BIJ | 396 | human insulin (N70/R30) | 300I.U (100I.U/mL) | Parenteral |  |
| 175330BIJ | 396 | insulin lispro | 300I.U (100I.U/mL) | Parenteral |  |
| 175332BIJ | 396 | Insulin lispro (insulin lispro protamine/insulin lispro 75%/25%) | 300I.U (100I.U/mL) | Parenteral |  |
| 175333BIJ | 396 | insulin lispro (insulin lispro protamine/insulin lispro 50%/50%) | 300I.U (100I.U/mL) | Parenteral |  |
| 441330BIJ | 396 | insulin aspart | 300I.U (100I.U/mL) | Parenteral |  |
| 441332BIJ | 396 | insulin aspart (insulin aspart protamine/insulin aspart 70%/30%) | 300I.U (100I.U/mL) | Parenteral |  |
| 441333BIJ | 396 | insulin aspart (insulin aspart protamine/insulin aspart 50%/50%) | 300I.U (100I.U/mL) | Parenteral |  |
| 461830BIJ | 396 | insulin glargine | as human insulin 300I.U (100I.U/mL) | Parenteral |  |
| 461832BIJ | 396 | insulin glargine | as human insulin 450I.U (300I.U/mL) | Parenteral |  |
| 484930BIJ | 396 | insulin glulisine | 10.47mg (3.49mg/mL) | Parenteral |  |
| 488730BIJ | 396 | insulin detemir | 42.6mg (14.2mg/mL) | Parenteral |  |
| 626700BIJ | 396 | insulin aspart | 90I.U (30I.U/mL) | Parenteral |  |
| 626700BIJ | 396 | insulin degludec | 210I.U (70I.U/mL) | Parenteral |  |
| 626830BIJ | 396 | Insulin degludec | 300I.U (100I.U/mL) | Parenteral |  |
| 666700BIJ | 396 | insulin glargine | as human insulin 300I.U (100I.U/mL) | Parenteral |  |
| 667000BIJ | 396 | insulin glargine | as human insulin 300I.U (100I.U/mL) | Parenteral |  |
| *<Everolimus>* | | | | |  |
| 485605ATB | 421 | everolimus | 5mg | Oral |  |
| 485606ATB | 421 | everolimus | 10mg | Oral |  |
| 485607ATB | 421 | everolimus | 2.5mg | Oral |  |
| *<Aromatase inhibitor>* | | | | |  |
| 109001ATB | 421 | anastrozole | 1mg | Oral |  |
| 182201ATB | 421 | letrozole | 2.5mg | Oral |  |
| *<CDK4/6 inhibitor>* | | | | |  |
| 655201ACH | 421 | palbociclib | 75mg | Oral |  |
| 655202ACH | 421 | palbociclib | 0.125g | Oral |  |
| 655203ACH | 421 | palbociclib | 0.1g | Oral |  |
| 686601ATB | 421 | abemaciclib | 50mg | Oral |  |
| 686602ATB | 421 | abemaciclib | 0.1g | Oral |  |
| 686603ATB | 421 | abemaciclib | 0.15g | Oral |  |
| *<Cytotoxic chemotherapy>* | | | | |  |
| 149430BIJ | 617 | doxorubicin hydrochloride | 10mg (2mg/mL) | Parenteral |  |
| 149432BIJ | 617 | doxorubicin hydrochloride | 50mg (2mg/mL) | Parenteral |  |
| 149433BIJ | 617 | doxorubicin hydrochloride | 0.1g (2mg/mL) | Parenteral |  |
| 152730BIJ | 617 | epirubicin hydrochloride | 10mg (2mg/mL) | Parenteral |  |
| 152731BIJ | 617 | epirubicin hydrochloride | 50mg (2mg/mL) | Parenteral |  |
| 148340BIJ | 421 | docetaxel | 0.12g (20mg/mL) | Parenteral |  |
| 148344BIJ | 421 | docetaxel | 20mg (20mg/mL) | Parenteral |  |
| 148348BIJ | 421 | docetaxel | 80mg (20mg/mL) | Parenteral |  |
| 148306BIJ | 421 | docetaxel | 0.15g (40mg/mL) | Parenteral |  |
| 148346BIJ | 421 | docetaxel | 20mg (40mg/mL) | Parenteral |  |
| 148350BIJ | 421 | docetaxel | 80mg (40mg/mL) | Parenteral |  |
| 148351BIJ | 421 | docetaxel | 0.12g (40mg/mL) | Parenteral |  |
| 148341BIJ | 421 | docetaxel | 20mg | Parenteral |  |
| 148342BIJ | 421 | docetaxel | 80mg | Parenteral |  |
| 207830BIJ | 421 | paclitaxel | 30mg (6mg/mL) | Parenteral |  |
| 207831BIJ | 421 | paclitaxel | 0.1g (6mg/mL) | Parenteral |  |
| 207832BIJ | 421 | paclitaxel | 0.15g (6mg/mL) | Parenteral |  |
| 207833BIJ | 421 | paclitaxel | 0.2g (6mg/mL) | Parenteral |  |
| 207835BIJ | 421 | paclitaxel | 0.3g (6mg/mL) | Parenteral |  |
| 122701ATB | 421 | capecitabine | 0.15g | Oral |  |
| 122702ATB | 421 | capecitabine | 0.5g | Oral |  |
| 164930BIJ | 421 | gemcitabine hydrochloride | as gemcitabine 1g (38mg/mL) | Parenteral |  |
| 164931BIJ | 421 | gemcitabine hydrochloride | as gemcitabine 0.2g (38mg/mL) | Parenteral |  |
| 164932BIJ | 421 | gemcitabine hydrochloride | as gemcitabine 2g (38mg/mL) | Parenteral |  |
| 248230BIJ | 421 | vinorelbine tartrate | as vinorelbine 10mg (10mg/mL) | Parenteral |  |
| 248231BIJ | 421 | vinorelbine tartrate | as vinorelbine 50mg (10mg/mL) | Parenteral |  |
| 621330BIJ | 421 | eribulin mesylate | as eribulin 0.88mg (0.44mg/mL) | Parenteral |  |

MFDS, Ministry of Food and Drug Safety
