# Supplementary material for: Statin use in patients with hormone receptor‐positive metastatic breast cancer treated with everolimus and exemestane
Source: Cancer Med. 2022 Oct 19;12(5):5461–70. doi: 10.1002/cam4.5369 (PMC10028110; doi:10.1002/cam4.5369)
Supplement: Supplementary file 3 — Table S2. [file CAM4-12-5461-s004.docx]

**Table S2. Duration of statin use based on EverX treatment period**

| Duration | Months, median (IQR) |
| --- | --- |
| Overall duration of statin use during study period (n=500) | 59.43 (24.97-92.71) |
| - Duration of overlapping with everolimus treatment (n=500) | 5.36 (2.63-11.27) |
| - Duration of statin use before everolimus treatment (n=388) | 55.74 (27.71-82.40) |
| - Duration of statin use after everolimus treatment (n=382) | 6.65 (2.10-17.91) |

EverX, everolimus and exemestane; n, number; IQR, interquartile range
