# Supplementary material for: Statin use in patients with hormone receptor‐positive metastatic breast cancer treated with everolimus and exemestane
Source: Cancer Med. 2022 Oct 19;12(5):5461–70. doi: 10.1002/cam4.5369 (PMC10028110; doi:10.1002/cam4.5369)
Supplement: Supplementary file 4 — Table S3. [file CAM4-12-5461-s002.docx]

**Table S3. Prior cytotoxic chemotherapeutic agents that patients received in this study**

| Chemo-agent | N (%)  (total n = 1,244) |
| --- | --- |
| Capecitabine | 218 (17.5%) |
| Docetaxel | 740 (59.5%) |
| Doxorubicin | 1000 (80.4%) |
| Epirubicin | 107 (8.6%) |
| Gemcitabine | 138 (11.1%) |
| Paclitaxel | 393 (31.6%) |
| Vinorelbine | 91 (7.3%) |
| Eribulin | 95 (7.6%) |

n, number
