# Supplementary material for: Statin use in patients with hormone receptor‐positive metastatic breast cancer treated with everolimus and exemestane
Source: Cancer Med. 2022 Oct 19;12(5):5461–70. doi: 10.1002/cam4.5369 (PMC10028110; doi:10.1002/cam4.5369)
Supplement: Supplementary file 5 — Table S4. [file CAM4-12-5461-s005.docx]

**Table S4. Baseline characteristics of the study population**

| **Variables** | **Never-statin group**  **(n = 988)** | **Ex-statin group**  **(n = 261)** | **Statin group**  **(n = 500)** | ***p*** |  |
| --- | --- | --- | --- | --- | --- |
| Age at starting EverX (yrs, median, IQR) | 54 (49-61) | 60 (54-67) | 61 (55-68) | <0.001 |  |
| ≥ 60 yrs | 295 (29.9%) | 138 (52.9%) | 291 (58.2%) | <0.001 |  |
| < 60 yrs | 683 (69.1%) | 123 (47.1%) | 209 (41.8%) |  |  |
| Prior AI treatment before EverX | 926 (93.7%) | 253 (96.9%) | 477 (95.4%) | 0.085 |  |
| Duration of prior AI treatment (mon, median, range) | 11.06 (4.90-22.67) | 14.65 (6.77-29.04) | 18.17  (8.41-30.49) | <0.001 |  |
| > 1 yr | 429 (46.3%) | 152 (60.1%) | 312 (65.4%) | <0.001 |  |
| > 6 mon, ≤ 1 yr | 206 (22.2%) | 43 (17.0%) | 73 (15.3%) |  |  |
| ≤ 6 mon | 291 (31.4%) | 58 (22.9%) | 92 (19.3%) |  |  |
| No. of cytotoxic chemotherapy before EverX | 712 (72.1%) | 193 (73.9%) | 339 (67.8%) | 0.127 |  |
| ≤ 2 | 636 (64.4%) | 173 (66.3%) | 319 (63.8%) | 0.034 |  |
| ≥ 3 | 76 (7.7%) | 20 (7.7%) | 20 (4.0%) |  |  |
| No. of cytotoxic chemotherapy after EverX | 567 (57.4%) | 135 (51.7%) | 269 (53.8%) | 0.172 |  |
| ≤ 2 | 379 (38.4%) | 90 (34.5%) | 179 (35.8%) | 0.474 |  |
| ≥ 3 | 168 (17.0%) | 45 (17.2%) | 90 (18.0%) |  |  |
| Co-morbidity |  |  |  |  |  |
| Hypertension | 261 (26.4%) | 144 (55.2%) | 325 (65.0%) | <0.001 |  |
| Diabetes mellitus (DM) | 208 (21.1%) | 108 (41.4%) | 311 (62.2%) | <0.001 |  |
| Existing DM | 138 (66.3%) | 81 (75.0%) | 203 (65.3%) | 0.167 |  |
| De novo DM | 70 (33.7%) | 27 (25.0%) | 105 (34.7%) |  |  |
| Hyperlipidemia | 308 (31.2%) | 206 (78.9%) | 442 (88.4%) | <0.001 |  |
| Timing of statin administration |  |  |  |  |  |
| Before EverX |  | 226 (86.6%) |  |  |  |
| After EverX |  | 35 (13.4%) |  |  |  |
| Duration of statin use (month, median, IQR) |  | 5.49 (1.87-25) | 59.43 (24.97-92.71) |  |  |

n, number; EverX, everolimus and exemestane; yrs, years; IQR, interquartile range; AI, aromatase inhibitor; yr, year; mon, months
