# Supplementary material for: Statin use in patients with hormone receptor‐positive metastatic breast cancer treated with everolimus and exemestane
Source: Cancer Med. 2022 Oct 19;12(5):5461–70. doi: 10.1002/cam4.5369 (PMC10028110; doi:10.1002/cam4.5369)
Supplement: Supplementary file 6 — Table S5. [file CAM4-12-5461-s001.docx]

**Table S5. Cox proportional hazard analysis for TTD for EverX in diabetic patients.**

| **Covariates** | **Univariable** | | | **Multivariable** | | |
| --- | --- | --- | --- | --- | --- | --- |
|  | **HR** | **95% CI** | ***p*** | **HR** | **95% CI** | ***p*** |
| Age at starting EverX |  |  |  |  |  |  |
| ≥ 60 yrs | Ref |  |  | Ref |  |  |
| < 60 yrs | 0.787 | 0.664-0.934 | 0.006 | 0.817 | 0.678-0.984 | 0.033 |
| Duration of prior AI treatment |  |  |  |  |  |  |
| > 1 yr | Ref |  |  | Ref |  |  |
| > 6 mon, ≤ 1 yr | 1.053 | 0.832-1.332 | 0.667 | 1.021 | 0.805-1.295 | 0.863 |
| ≤ 6 mon | 1.119 | 0.900-1.391 | 0.311 | 1.087 | 0.870-1.357 | 0.464 |
| Prior cytotoxic chemotherapy |  |  |  |  |  |  |
| No | Ref |  |  | Ref |  |  |
| Yes | 1.402 | 1.136-1.730 | 0.002 | 1.399 | 1.122-1.744 | 0.003 |
| Hypertension |  |  |  |  |  |  |
| No | Ref |  |  | Ref |  |  |
| Yes | 1.126 | 0.946-1.340 | 0.183 | 1.088 | 0.899-1.317 | 0.385 |
| Diabetes mellitus |  |  |  |  |  |  |
| Existing DM | Ref |  |  | Ref |  |  |
| De novo DM | 0.571 | 0.476-0.684 | <0.001 | 0.617 | 0.511-0.745 | <0.001 |
| Hyperlipidemia |  |  |  |  |  |  |
| No | Ref |  |  | Ref |  |  |
| Yes | 0.816 | 0.679-0.981 | 0.030 | 0.84 | 0.676-1.043 | 0.115 |
| Use of statin |  |  |  |  |  |  |
| No | Ref |  |  | Ref |  |  |
| Yes | 0.713 | 0.602-0.844 | <0.001 | 0.813 | 0.668-0.991 | 0.040 |
| Use of metformin |  |  |  |  |  |  |
| No | Ref |  |  | Ref |  |  |
| Yes | 0.734 | 0.620-0.869 | <0.001 | 0.724 | 0.602-0.870 | <0.001 |
| Use of insulin |  |  |  |  |  |  |
| No | Ref |  |  | Ref |  |  |
| Yes | 1.140 | 0.963-1.349 | 0.128 | 1.152 | 0.957-1.386 | 0.134 |

TTD, time to treatment duration; EverX, everolimus and exemestane; HR, hazard ratio; CI, confidence interval; yrs, years; Ref, reference; AI, aromatase inhibitor; yr, year; mon, months
